# Obesity and periodontal diseases: A review

**DOI:** 10.6026/973206300220392

**Published:** 2026-01-31

**Authors:** Naif Alwithanani

**Affiliations:** 1 Department of Oral and Maxillofacial Surgery and Diagnostic science Faculty of dentistry, Taif University, Taif, Saudi Arabia

**Keywords:** Obesity, periodontitis, inflammation, adipokines, metabolic syndrome

## Abstract

Obesity and periodontal diseases are highly prevalent chronic conditions linked by shared inflammatory and metabolic pathways. Obesity-induced systemic inflammation, adipokines imbalance and oxidative stress can exacerbate periodontal breakdown, while epidemiological evidence demonstrates a consistent association between excess adiposity and increased periodontitis risk. Understanding these mechanisms supports integrated management strategies combining periodontal therapy with lifestyle and metabolic interventions. This review summarises current biological, epidemiological and clinical evidence on the obesity-periodontitis relationship.

## Background:

Obesity has emerged as one of the most significant non-communicable health challenges of the 21st century, affecting more than 1.9 billion adults worldwide, of whom over 650 million are classified as obese according to the World Health Organization [1, 2-3]. The prevalence has risen steadily across both developed and developing regions, driven by urbanisation, sedentary behaviour and dietary transitions. India has also witnessed a substantial increase in overweight and obesity, with recent NFHS-5 data reporting a notable rise among adults aged 18-49 years [4]. Obesity is now well recognised as a chronic, low-grade inflammatory disorder characterised by excess adiposity, metabolic dysfunction and altered secretion of adipokines, all of which have systemic effects [3, 5]. Periodontal diseases represent another major public health burden globally, contributing significantly to tooth loss, impaired quality of life and increased healthcare costs. The Global Burden of Disease (GBD) 2019 analysis identified severe periodontitis as the 11th most prevalent condition worldwide, affecting approximately 796 million individuals [2, 6]. Periodontitis is a multifactorial inflammatory disease driven by dysbiotic biofilms and an exacerbated host immune response, ultimately leading to connective tissue breakdown and alveolar bone loss [7, 8]. Growing evidence suggests a biologically plausible relationship between obesity and periodontal diseases. Numerous epidemiological studies and meta-analyses have also documented a consistent association, with obese individuals demonstrating a higher risk and severity of periodontitis [9, 10, 11- 12]. Shared pathways such as chronic systemic inflammation, oxidative stress, insulin resistance and adipokines dysregulation have been implicated in linking excess body fat with enhanced periodontal tissue destruction [6, 13]. Given the rising global burden of conditions and their interrelated pathophysiology, understanding the obesity-periodontitis link is essential for holistic patient care. Therefore, it is of interest to review synthesises current evidence on biological mechanisms, epidemiological trends and clinical implications to guide integrated prevention and management strategies.

## Pathophysiology of obesity:

Obesity is increasingly recognised as a complex, chronic, relapsing metabolic disorder characterised by excessive accumulation of adipose tissue and systemic physiological alterations. Beyond its traditional role as an energy reservoir, adipose tissue functions as an active endocrine organ that secretes a wide array of adipokines, cytokines and metabolites influencing whole-body homeostasis [3]. In individuals with obesity, expansion of adipose tissue occurs through both hypertrophy and hyperplasia of adipocytes, accompanied by hypoxia, mitochondrial dysfunction and extracellular matrix remodelling. These changes promote infiltration of macrophages and other immune cells, contributing to a persistent state of low-grade systemic inflammation often termed "metaflammation" [6]. Dysregulated adipokine production plays a central role in the pathophysiology of obesity. Leptin levels are markedly elevated but are often associated with leptin resistance, impairing appetite regulation and metabolic signalling. Conversely, adiponectin - a key anti-inflammatory and insulin-sensitising molecule - is significantly reduced in obesity, promoting metabolic dysfunction [5]. Other pro-inflammatory mediators such as tumour necrosis factor-α (TNF-α), interleukin-6 (IL-6) and resistin are overexpressed, further amplifying immune activation and contributing to systemic inflammatory load. Insulin resistance is another critical element linking obesity to its multisystem complications. Chronic exposure to elevated free fatty acids and inflammatory cytokines disrupts insulin signalling pathways, impairing glucose uptake and favouring hyperglycaemia. This mechanism underlies the strong association of obesity with metabolic syndrome, type 2 diabetes, cardiovascular diseases and related endocrine disturbances [3]. Moreover, oxidative stress is heightened in obesity due to increased reactive oxygen species generation and reduced antioxidant capacity. This contributes to endothelial dysfunction, tissue damage and heightened vulnerability to chronic inflammatory diseases. Obesity also alters immune cell phenotypes, skewing macrophages toward pro-inflammatory M1 states and disrupting neutrophil and lymphocyte functions.

## Pathogenesis and classification of periodontal diseases:

Periodontal diseases encompass a group of inflammatory conditions affecting the supporting structures of the teeth, primarily driven by interactions between microbial biofilms and the host immune response. The updated 2017 AAP/EFP classification redefined periodontitis as a multifactorial, chronic inflammatory disease characterised by progressive destruction of periodontal ligament, cementum and alveolar bone. This framework categorises periodontitis based on staging (severity and complexity) and grading (rate of progression and risk factors), providing a more comprehensive approach for diagnosis and management [8]. The pathogenesis of periodontal disease begins with accumulation of bacterial biofilms at the gingival margin. Key periodontal pathogens such as Porphyromonas gingivalis, Tannerella forsythia and Treponema denticola contribute to microbial dysbiosis, triggering innate immune activation. Pattern-recognition receptors on gingival epithelial cells and neutrophils recognise microbial components, leading to release of inflammatory mediators including interleukin-1β (IL-1β), IL-6, prostaglandin E2 and tumour necrosis factor-α (TNF-α) [7, 14, 15]. This inflammatory cascade promotes vasodilation, connective tissue degradation and recruitment of immune cells. Chronic inflammation leads to overproduction of matrix metalloproteinases (MMPs), osteoclast activation via receptor activator of nuclear factor-κB ligand (RANKL) and ultimately, alveolar bone resorption. The transition from gingivitis to periodontitis is influenced not only by microbial virulence but also by host susceptibility factors including systemic inflammation, metabolic abnormalities, genetics, smoking and diabetes mellitus [16]. Given these complex interactions, periodontal disease is now widely acknowledged as an immunoinflammatory condition rather than a purely infectious process. The host's exaggerated inflammatory response, rather than bacterial load alone, determines disease severity and progression [7]. This conceptual shift aligns closely with increasing evidence linking systemic conditions-such as obesity and metabolic syndrome - to heightened periodontal destruction. Understanding the multifactorial nature and biological mechanisms underpinning periodontitis is critical for evaluating how systemic disorders like obesity can modify host responses and increase vulnerability to periodontal tissue breakdown.

## Biological links between obesity and periodontal diseases:

The association between obesity and periodontal diseases is supported by a growing body of mechanistic evidence demonstrating common inflammatory pathways, metabolic disturbances and immunological alterations. Obesity induces a chronic state of low-grade systemic inflammation - often termed metaflammation - characterised by increased production of pro-inflammatory cytokines, adipokines and acute- phase reactants [6]. These systemic mediators have direct and indirect effects on periodontal tissues, amplifying host inflammatory responses to bacterial biofilms and accelerating periodontal breakdown. A key mechanism linking obesity to periodontitis is dysregulation of adipokines. Hypertrophic adipose tissue secretes elevated levels of leptin, resistin, tumour necrosis factor-α (TNF-α) and interleukin-6 (IL-6), while anti-inflammatory adiponectin levels are markedly reduced [5]. These adipokine imbalances contribute to heightened immune activation, oxidative stress and impaired tissue repair. Elevated systemic TNF-α and IL-6 promote activation of matrix metalloproteinases (MMPs), osteoclast differentiation and receptor activator of nuclear factor-κB ligand (RANKL) production-key mediators of alveolar bone resorption [7]. Reduced adiponectin, which normally inhibits inflammatory cytokine release, further exacerbates periodontal tissue vulnerability [13]. Obesity-associated insulin resistance also plays a significant role. Chronic hyperinsulinaemia impairs neutrophil chemotaxis and phagocytic function, disrupts fibroblast activity and increases endothelial dysfunction, thereby compromising periodontal tissue integrity [14]. Insulin resistance increases oxidative stress and impairs microvascular blood flow, limiting nutrient transport and immune cell recruitment essential for periodontal maintenance and healing. These mechanisms mirror the well-established effects of type 2 diabetes on periodontal destruction, underscoring the metabolic links between obesity and oral health [17, 18]. In addition, obesity influences the composition and behaviour of oral and gut microbiota. Evidence suggests that obesity-related shifts in microbial communities may contribute to enhanced systemic inflammation and exaggerated immune responses in the periodontium [19]. Altered gut microbiota increases intestinal permeability, enabling translocation of endotoxins such as lipopolysaccharides (LPS) into circulation. Elevated circulating LPS levels trigger Toll-like receptor (TLR)-mediated inflammatory responses, which amplify periodontal inflammation in susceptible individuals [20]. Oxidative stress is another central pathway. Obesity increases reactive oxygen species (ROS) generation and reduces antioxidant defences, resulting in oxidative damage to periodontal fibroblasts, epithelial cells and extracellular matrix components. This environment favours periodontal pathogen proliferation and tissue breakdown [15]. Collectively, these biological mechanisms form a strong foundation for understanding how obesity may enhance both susceptibility to and severity of periodontal diseases. Rather than functioning as an isolated oral condition, periodontitis should be viewed within the broader context of systemic inflammation and metabolic dysregulation. Clarifying these mechanisms is essential for guiding targeted prevention and treatment approaches in obese individuals.

## Epidemiological evidence:

The epidemiological link between obesity and periodontal diseases has been extensively explored over the past two decades, with a strong and consistent association documented across cross-sectional, case-control and longitudinal studies. Early population-based investigations demonstrated that individuals with elevated body mass index (BMI), waist circumference, or increased body fat percentage were more likely to exhibit signs of periodontal destruction, including attachment loss and deeper periodontal pockets [18]. These associations persisted after adjusting for major confounders such as age, smoking and socioeconomic status, suggesting an independent contribution of obesity to periodontal risk. Several systematic reviews and meta-analyses have strengthened the evidence base. A Research analysed data from 70 studies and reported that obese individuals had a significantly higher odds of periodontitis compared to normal- weight individuals (pooled OR ≈ 1.3-1.8) [9]. Similarly, research found that obesity was associated not only with increased prevalence but also with greater severity of periodontal disease indicators, including gingival inflammation, bleeding on probing and radiographic bone loss [10, 11]. More recent evidence reaffirmed these findings, indicating that excess body fat increases the risk of periodontitis by up to 35% [12]. Longitudinal studies provide further insight into the temporal relationship between obesity and periodontal disease progression. Data from cohort studies in Japan, the United States and Scandinavian countries consistently show that higher BMI and central adiposity predict greater periodontal attachment loss and tooth loss over follow-up periods ranging from five to ten years [16]. These findings support the notion that obesity is not merely correlated with periodontal disease but may contribute to its development and progression. The epidemiological burden appears to vary across demographic groups. Younger adults with obesity show early manifestations of gingival inflammation and early periodontal changes, suggesting that excessive adiposity may accelerate disease onset. Sex-specific trends have also emerged: women with obesity, especially those with metabolic syndrome, appear to exhibit stronger associations with periodontal destruction, possibly due to hormonal and inflammatory differences. Additionally, periodontal risk increases markedly when obesity coexists with diabetes, smoking, or hypertension, indicating synergistic effects of metabolic and behavioural risk factors. Evidence from low- and middle-income countries (LMICs), including India, aligns with global findings. Studies reflect a parallel rise in obesity and periodontitis prevalence, with urban populations demonstrating particularly strong associations [4]. As dietary transitions and sedentary lifestyles become more common in LMICs, the obesity-periodontitis nexus is expected to intensify.

## Clinical implications:

The clinical implications of the relationship between obesity and periodontal diseases are increasingly recognised in contemporary dental practice. Given that obesity induces systemic inflammation, oxidative stress and metabolic disturbances, its presence can significantly influence periodontal health, disease progression and response to therapy. Several clinical studies have demonstrated that individuals with obesity exhibit greater gingival inflammation, deeper periodontal pockets and accelerated connective tissue breakdown compared with normal-weight individuals [18]. These findings underscore the need for clinicians to consider obesity as a significant modifying factor in periodontal risk assessment. Obesity has also been shown to adversely affect periodontal treatment outcomes. Studies reported that patients with obesity demonstrated a reduced clinical response to nonsurgical periodontal therapy, including limited improvements in probing depth reduction and clinical attachment gain [17, 18]. This diminished therapeutic response is likely attributable to impaired immune regulation, delayed wound healing and reduced microvascular perfusion associated with excess adiposity. Additionally, insulin resistance-frequently observed in individuals with obesity-may further compromise periodontal tissue repair by altering fibroblast function and impairing neutrophil activity [14]. The coexistence of metabolic syndrome or type 2 diabetes in obese individuals further amplifies periodontal susceptibility. Hyperglycaemia enhances advanced glycation end-product (AGE) formation, stimulates pro-inflammatory cytokine production and disrupts collagen homeostasis, thereby exacerbating periodontal destruction. Thus, integrated management strategies that address both periodontal inflammation and metabolic dysregulation are essential for optimal clinical outcomes. From a preventive standpoint, dental practitioners should incorporate obesity screening into routine periodontal evaluation. Body mass index (BMI), waist circumference and lifestyle factors such as diet and physical activity should be recorded and considered when stratifying patient risk. Interdisciplinary collaboration with physicians, endocrinologists and nutritionists can facilitate comprehensive patient management. Evidence suggests that weight reduction, improved dietary patterns and increased physical activity may enhance periodontal treatment response and reduce systemic inflammatory burden [16].

## Role of diet, lifestyle and microbiome:

Diet, lifestyle behaviours and microbiome alterations represent critical factors linking obesity with periodontal diseases. Dietary patterns rich in saturated fats, refined carbohydrates and added sugars contribute significantly to excess caloric intake, adiposity and chronic inflammation. High-fat diets stimulate the release of pro-inflammatory cytokines such as TNF-α and IL-6, thereby intensifying systemic inflammatory load and potentially exacerbating periodontal tissue destruction [5]. Furthermore, frequent consumption of sugary foods promotes dysbiosis within the oral microbiota, facilitating the growth of pathogenic species associated with periodontal disease. Sedentary lifestyle behaviours further compound this risk. Physical inactivity reduces insulin sensitivity, increases oxidative stress and negatively influences immune function, all of which contribute to increased periodontal susceptibility. Studies indicate that individuals with more active lifestyles exhibit lower levels of systemic inflammatory biomarkers and demonstrate improved periodontal outcomes compared to those with sedentary habits [16]. The microbiome represents another crucial link in the obesity-periodontitis axis. Obesity is associated with significant shifts in both gut and oral microbial communities. Gut microbiota studies have shown reduced diversity and a characteristic increase in Firmicutes-to- Bacteroidetes ratios in individuals with obesity [19]. These alterations promote increased intestinal permeability, enabling translocation of lipopolysaccharides (LPS) into systemic circulation-a process known as metabolic endotoxemia. Circulating LPS can engage Toll-like receptor pathways, intensifying host inflammatory responses within periodontal tissues [20]. Similarly, oral microbiome dysbiosis may be influenced by dietary habits and metabolic status, contributing to an environment conducive to periodontal pathogen proliferation. Taken together, diet, lifestyle behaviours and microbiome alterations act synergistically to enhance both systemic inflammation and local periodontal breakdown.

## Management strategies:

Management of periodontal diseases in individuals with obesity requires an integrated approach that targets both local periodontal inflammation and the underlying systemic metabolic disturbances. Conventional periodontal therapy - comprising scaling and root planning, oral hygiene instruction and risk-factor modification - remains the cornerstone of treatment. However, evidence suggests that obese individuals may exhibit a less favourable response to nonsurgical therapy, including reduced probing depth reduction and clinical attachment gain, likely due to impaired wound healing, altered immune responses and endothelial dysfunction [17]. Therefore, clinicians should consider obesity as an important modifier of treatment planning and expected outcomes. Weight management is a critical adjunctive strategy. Studies indicate that intentional weight reduction improves metabolic parameters, reduces systemic inflammatory burden and may enhance periodontal treatment outcomes. Weight loss achieved through dietary modification, increased physical activity, or multidisciplinary obesity-management programmes has been associated with reductions in circulating inflammatory cytokines such as TNF-α and IL-6, which play key roles in periodontal tissue destruction [6]. In this context, dietary counselling-particularly promoting nutrient-dense, anti-inflammatory diets rich in fruits, vegetables and omega-3 fatty acids-can complement periodontal therapy by improving both systemic and oral health. Interdisciplinary collaboration is essential. Coordination between dentists, physicians, endocrinologists and nutritionists ensures comprehensive evaluation and management of metabolic comorbidities such as insulin resistance, type 2 diabetes and metabolic syndrome, all of which amplify periodontal risk [14]. Behavioural counselling to address smoking, alcohol intake and physical inactivity further strengthens periodontal outcomes. Emerging interventions, including probiotics, prebiotics and targeted modulation of the oral and gut microbiomes, offer additional avenues for improving periodontal and metabolic health. Although evidence is evolving, microbiome-focused therapies may help reduce systemic inflammation and restore microbial balance [19].

## Future research directions:

Although substantial evidence supports an association between obesity and periodontal diseases, further research is needed to clarify causal pathways, refine risk prediction and optimise clinical management. Longitudinal cohort studies with standardised diagnostic criteria and repeated periodontal measurements are essential for determining the temporal sequence and identifying individuals at highest risk [9]. Integrating advanced biomarkers such as adipokine profiles, oxidative stress markers and inflammatory mediators may enhance understanding of mechanistic pathways linking excess adiposity to periodontal destruction [5, 6]. Emerging fields such as genomics, metabolomics and microbiome science offer valuable opportunities for precision medicine. Multi-omics studies could elucidate individual variations in immune responses, microbial dysbiosis and metabolic signatures that mediate susceptibility to periodontal diseases in obese populations [19]. Clinical trials evaluating the impact of weight reduction, dietary modification, anti- inflammatory therapies and microbiome-targeted interventions on periodontal outcomes are also needed to inform evidence-based recommendations. Future research should additionally explore the synergistic influence of obesity with other systemic conditions such as diabetes, hypertension and metabolic syndrome, particularly in diverse populations where the burden of both diseases continues to rise. Such efforts will support development of integrated, personalised strategies for prevention and management.

## Conclusion:

Obesity and periodontitis share inflammatory and metabolic mechanisms, with obesity increasing systemic inflammation and worsening periodontal breakdown. Evidence shows higher risk, greater severity and reduced treatment response in obese individuals. Effective management requires integrating periodontal therapy with weight control, metabolic management and lifestyle modification to improve overall outcomes.
